# Multi UAV Coverage Path Planning in Urban Environments

**DOI:** 10.3390/s21217365

**Published:** 2021-11-05

**Authors:** Javier Muñoz, Blanca López, Fernando Quevedo, Concepción A. Monje, Santiago Garrido, Luis E. Moreno

**Affiliations:** Robotics Lab, Universidad Carlos III de Madrid, Av. Madrid 30, 28911 Leganés, Spain; bllopezp@ing.uc3m.es (B.L.); fquevedo@ing.uc3m.es (F.Q.); cmonje@ing.uc3m.es (C.A.M.); sgarrido@ing.uc3m.es (S.G.); moreno@ing.uc3m.es (L.E.M.)

**Keywords:** UAVs, coverage path planning, path planning, fast marching

## Abstract

Coverage path planning (CPP) is a field of study which objective is to find a path that covers every point of a certain area of interest. Recently, the use of Unmanned Aerial Vehicles (UAVs) has become more proficient in various applications such as surveillance, terrain coverage, mapping, natural disaster tracking, transport, and others. The aim of this paper is to design efficient coverage path planning collision-avoidance capable algorithms for single or multi UAV systems in cluttered urban environments. Two algorithms are developed and explored: one of them plans paths to cover a target zone delimited by a given perimeter with predefined coverage height and bandwidth, using a boustrophedon flight pattern, while the other proposed algorithm follows a set of predefined viewpoints, calculating a smooth path that ensures that the UAVs pass over the objectives. Both algorithms have been developed for a scalable number of UAVs, which fly in a triangular deformable leader-follower formation with the leader at its front. In the case of an even number of UAVs, there is no leader at the front of the formation and a virtual leader is used to plan the paths of the followers. The presented algorithms also have collision avoidance capabilities, powered by the Fast Marching Square algorithm. These algorithms are tested in various simulated urban and cluttered environments, and they prove capable of providing safe and smooth paths for the UAV formation in urban environments.

## 1. Introduction

Unmanned Aerial Vehicles (UAVs), due to their great versatility, have been recently used for tasks such as smart farming [1], surveillance [2,3,4], wildfire control [5,6], delivery [7,8,9], and natural disaster situations [10,11] among others. UAVs are mainly classified into two categories: fixed-wing and rotary wing. Fixed-wing UAVs have rigid wings which allow flying based on the lift created by forward speed, while rotary-wing UAVs use rotary blades to fly. Rotary-wing UAVs are more versatile since they allow vertical take off and landing, great maneuverability, low-altitude flights, and hovering tasks [12].

The Coverage Path Planning (CPP) problem is considered a motion planning subtopic, which objective is to find a path which covers every point of a certain area of interest while avoiding obstacles [13]. This area of interest is commonly decomposed into cells of different sizes and resolutions, based on the computation capabilities available or the UAV’s sensors range, which commonly are LiDAR sensors or cameras. The Coverage Path Planning problem is usually divided into two main categories. One category consists of finding the optimal path that covers a predefined target area, while the other one consists of calculating a smooth path that connects every point in the given set of designated waypoints.

For the CPP category, which focuses on covering a target area, the first step is to consider the parameters that define the area. The perimeter of the target area can be represented by a series of vertices, where each vertex is defined by a pair of coordinates and its internal angle. Once the target area is defined, it is necessary to apply some kind of cellular decomposition technique to ensure the complete coverage [13]. The most common cellular decomposition methods used for the CPP problem are exact and approximate cellular decomposition.

**Figure 1:**
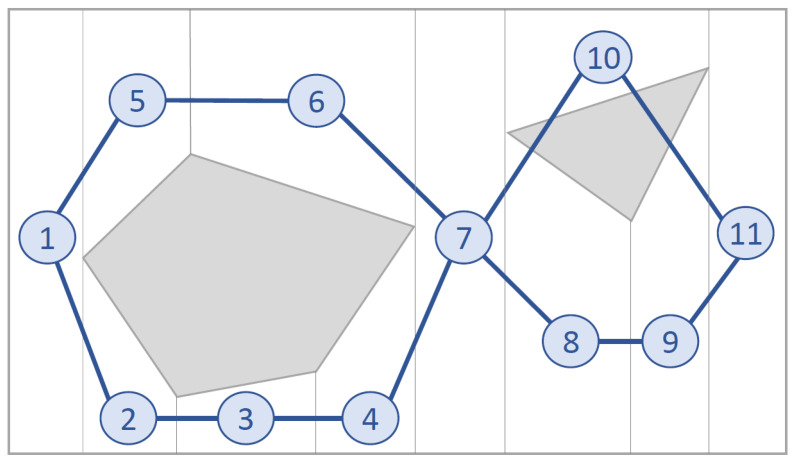
Sub-areas obtained from exact cellular decomposition [12]. Reprinted from ref. [12].

Exact cellular decomposition techniques divide the target area into sub-areas, which are usually covered using back-and-forth motions. Following this division, the CPP problem can be considered as a graph search to create a path that connects all sub-areas. Therefore, the final path is a combination of the back-and-forth motions inside each sub-area and the path that connects adjacent sub-areas [14]. An example of exact cellular decomposition can be seen in Figure 1.

Some of the CPP works that use the exact cellular decomposition technique are: coverage of concave polygonal areas by decomposing them into non-concave sub areas and using back-and-forth motions [15,16,17], coverage for fixed-wing UAVs analyzing wind to decrease flight time [18,19], optimal coverage for fixed-wing UAVs able to avoid obstacles with arbitrary shapes [20,21], spiral and back-and-forth motions in concave areas [22], a cooperative strategy to simultaneously cover sub-areas of the given target area by a team of UAVs [23], and a spiral CPP algorithm for missions in coastal regions using multiple heterogeneous UAVs [24,25], among others.

**Figure 2:**
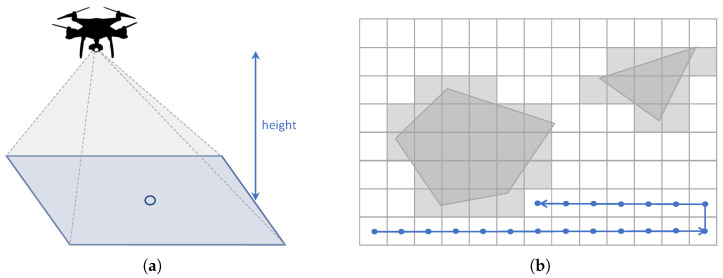
(**a**) Projected area of the UAV; (**b**) Cells obtained by applying approximate cellular decomposition [12]. Reprinted from ref. [12].

Approximate cellular decomposition techniques divide the target area into regular cells [13]. These cells usually have a square form, and are commonly used to generate coverage paths [14]. The size of the cells is commonly determined by the field of view (FOV) of the sensor, usually a LiDAR sensor or a camera, attached to the UAV and the distance from the UAV to the terrain as seen in Figure 2.

A few CPP articles that feature approximate cellular decomposition techniques are: an approach for image mosaicing in precision agriculture with irregular-shaped areas [26], an optimal CPP algorithm where the area is represented by points of interest [27], another approach for precision agriculture that uses a team of heterogeneous quadcopters [28], a meta-heuristic algorithm named Harmony Search (HS) [29], online non-uniform CPP using different grid sizes [30,31], high-resolution aerial sensing with multiple heterogeneous UAVs for non-convex areas [32], pheromone-based methods [33], and a combination of digital pheromones and evolutionary strategies for coordination of multiple UAVs [34], among others.

Other CPP approaches use waypoints to define the targets to cover: [35] uses waypoints for 3D CPP for autonomous structural inspection operations using aerial robots and [27] develops an optimal CPP algorithm where the area is represented by points of interest.

Most approaches focus on calculating coverage paths and optimizing the computation time and the time needed to cover the area, but they usually only consider empty surfaces with no obstacles. However, there exist some methods that take into account coverage areas with obstacles [26,36,37]. The main disadvantage of these works compared to our method, is that they usually decompose the area to cover into big cells, and completely avoid the cells that contain any obstacles.

More recent publications present different approaches for planning CPP missions. Muñoz et al. [38] propose a Differential Evolution-based CPP method for search and rescue tasks in marine environments. Chen et al. [39] propose a clustering-based CPP method, which consists in dividing the target area into clusters and assign each area to a different UAV based on the area properties and the UAV’s scanning width and flying speed. However, this work can be seen as a task assignment method, and could be used as a previous step to our proposed target area CPP method. Cho et al. [40] highlight a two-phase method for solving the CPP problem of multiple-UAV areas in maritime search and rescue. The first phase decomposes the search area into a graph, and the second phase uses the mixed-integer linear programming (MILP) to calculate an optimal path that minimizes the completion time. Melo et al. [41] introduce a 3D dynamic algorithm for CPP capable of covering a set of 3D waypoints while allowing optimization of energy usage.

This work proposes two methods for multi UAV optimal CPP in urban environments that use the approximate cellular decomposition approach and have collision-avoidance capabilities. One method generates a coverage path given a target area using back-and-forth motions, while the other method creates a smooth path that covers a set of given waypoints. Furthermore, both methods are applied to teams of UAVs by developing an algorithm that follows a leader-follower approach [42], creating UAV formations. For the collision-avoidance capabilities and the UAV formation algorithm, the FM${}^{2}$ method is used. Since the flying of drones over cities is forbidden in Spain at the moment, the algorithms are tested in simulated urban environments.

The main advantage of the proposed algorithms over previous approaches, is that the UAVs fly as a formation instead of sub-dividing the target area. Furthermore, this formation can be deformed accordingly so it avoids any obstacles that may be in the way. The state of the art methods are really efficient when performing clustering and CPP, but cannot be applied to urban or cluttered environments that may contain obstacles such as communication towers, cranes, small buildings or skyscrapers.

The rest of this paper is organized as follows. Section 2 contains all the main aspects of the proposed solution to the coverage path planning problem for search operations. Section 3 analyses the overall performance of the strategy and evaluates its applicability to real world case scenarios. Section 4 provides a discussion about the main advantages of our proposal, and compares it with similar works in the literature. Lastly, Section 5 highlights the main conclusions and contributions drawn throughout the progress of this work.

## 2. Description of the Approach

The development of the algorithm has been divided into three major stages, which will be detailed hereinafter. Firstly, the method was used to achieve the leader-follower drone formation in order to avoid obstacles while keeping a sufficient safety distance among vehicles. Secondly, the process was followed to obtain the coverage path for the leader drone. Finally, the approach through which the follower UAVs obtain their path was based on the already computed collision-free leader path and the aforementioned leader-follower formation strategy. The proposed algorithm aims to be applied in both search target areas and in linear coverage missions, in which a series of waypoints must be visited by the vehicles. The second stage mentioned above varies slightly for these two scenarios and thus, both applications are explained separately in this section. It must be mentioned that in this project UAVs are considered as a point-size object flying through free airspace.

In this work, we only consider the planning for the coverage path exclusively, as we do not take into account the planification needed from the UAV depot to the start of the CPP mission, and back to the depot after the CPP mission has finished. The algorithm used to plan these paths and manage traffic in cluttered urban environments is shown in our previous publication [43].

### 2.1. UAV Formation Approach Using Fast Marching Square

**Figure 3:**
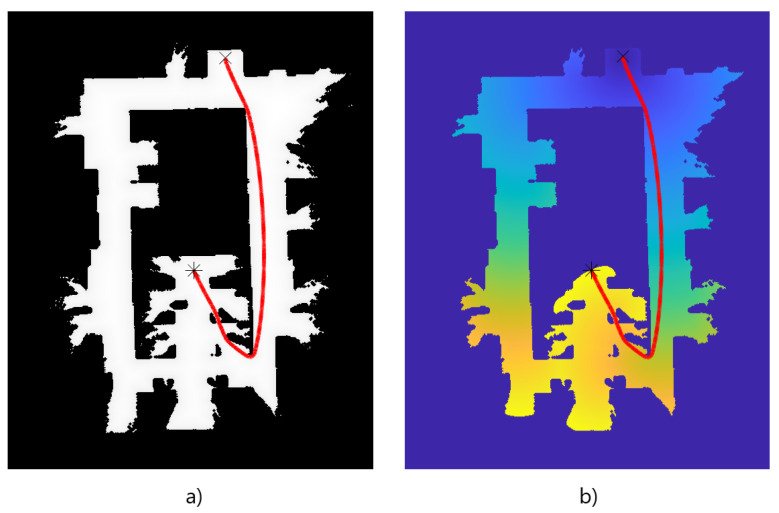
(**a**) initial binary map, (**b**) time of arrival of the propagating wavefront. The path obtained with the Fast Marching Method (FMM) is shown as a red line.

**Figure 4:**
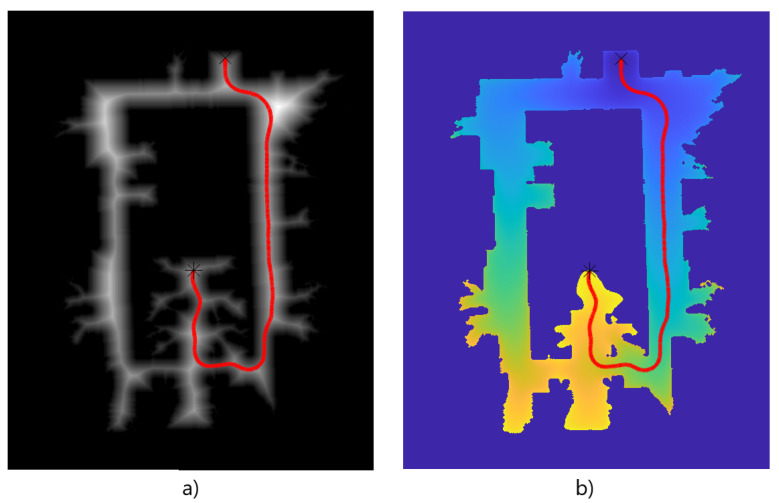
(**a**) velocity map, (**b**) time of arrival of the wavefront. The path obtained with Fast Marching Square (FM${}^{2}$) is shown as a red line.

Now that the basis for the CPP algorithms has been detailed, it is necessary to define the formation strategy that will be followed by the drones. In this case, a leader-follower approach is implemented, using a virtual leader in the case of an even number of drones. The drones follow a triangular formation, which can be deformed to avoid obstacles using the Fast Marching Square (FM${}^{2}$) method [44]. This path planning method is a variation of the original Fast Marching method [45]. It creates a velocity map given a binary map of occupation by propagating a wavefront taking all the obstacles as source points. To better illustrate the differences between the two methods, paths are calculated and shown in Figure 3 and Figure 4. Both figures show that the path obtained with the FM${}^{2}$ method is more smooth and reproducible by a robot, thanks to the calculation of the velocity map.

**Figure 5:**
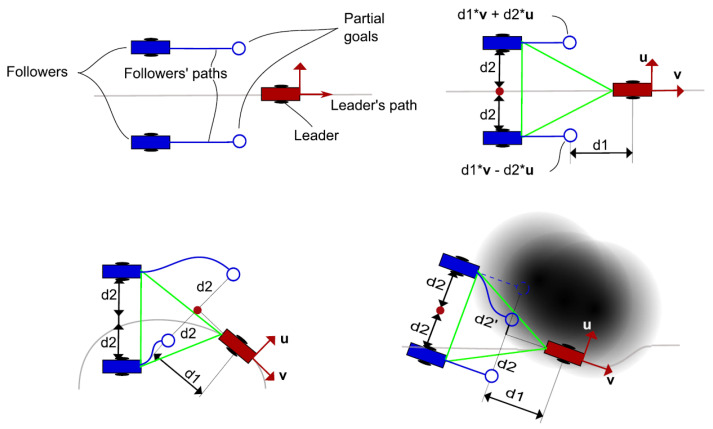
UAVs formation algorithm. From top left to bottom right: main components of the formation approach; reference definition of the triangle-shaped UAV formation; modification of the partial goals of the followers according to the leader position; modification of partial goals according to the obstacles of the environment.

The velocity map values range from 0 to 1, representing the maximum speed allowed for the drone. Obstacles will imply speeds equal to zero, whilst points in space far enough away from obstacles will allow maximum speeds. When computing a path for the vehicles to follow, FM${}^{2}$ method will get the shortest path from the initial position to the goal position that lets the vehicles navigate at higher speeds. Using this velocity map, the partial goals of the followers of the formation can be adjusted so obstacles are avoided safely. An example of this formation approach is shown in Figure 5.

For a given number of drones *n*, the desired positions in the formation are calculated as follows. First, a multiplier is applied to calculate the distance in the perpendicular axis to the leader’s path. If *n* is even, a virtual leader is used, whereas if *n* is odd, one of the drones is used as the leader. *i* is an auxiliary variable used to calculate the multiplier variable for each UAV.

(1) $$\begin{array}{cc} \; & \mathrm{If}\;n\;\mathrm{is}\;\mathrm{odd}\;\;\;\;\;\;\;\;multiplier\left(2i:2i+1\right)=i\;\;\;\;\;\;\;\;\;\;\;\;\;\;\;\;\;\mathrm{for}\;\;i=1:\left(\frac{n}{2}-0.5\right)\\ \; & \mathrm{If}\;n\;\mathrm{is}\;\mathrm{even}\;\;\;\;\;\;\;multiplier\left(2i:2i+1\right)=i-0.5\;\;\;\;\;\;\;\;\mathrm{for}\;\;i=1:\frac{n}{2} \end{array}$$

**Figure 6:**
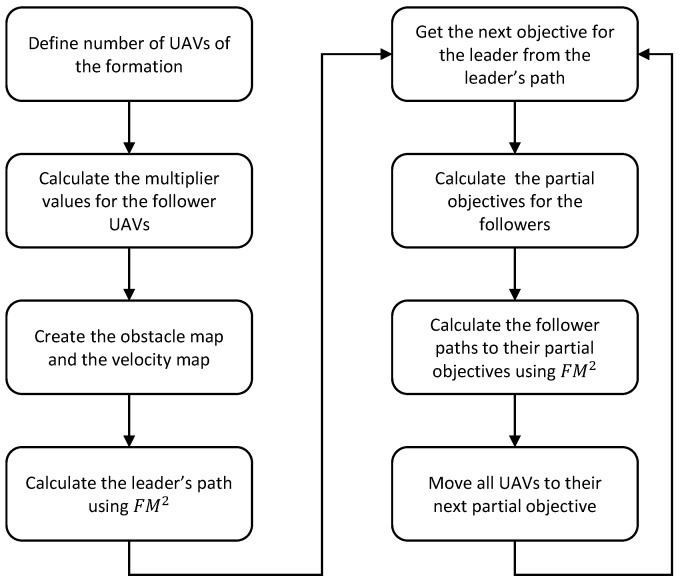
Flowchart for the FM${}^{2}$ formation strategy.

Now that the multiplier value for each drone is known, the next partial goals for the followers are calculated as follows:

(2) $$partialGoal\left(n\right)=leaderGoal-multiplier\left(n\right)d_{1}v\pm B\left(n\right)multiplier\left(n\right)d_{2}u$$

where partialGoal(n) is the next partial goal for drone *n*, leaderGoal is the next point in the leader’s path, B(n) is the velocity value at the position of drone *n*, $d_{1}$ and $d_{2}$ are the respective parallel and perpendicular distances to the leader’s path, and v and u are the vectors that define said parallel and perpendicular directions. Then, the UAVs are added as obstacles for the other UAVs in the team, and their paths from their current positions to their new partial goals are calculated using FM${}^{2}$. The steps of the algorithm are detailed in Algorithm 1 and in Figure 6.
**Algorithm 1** Routine of the FM${}^{2}$ formation strategy  1:Define number of UAVs of the formation  2:Calculate the multiplier values based on the number of UAVs following Equation (1)  3:Wo← Occupation matrix of the environment  4:*W*← Velocity map computed from Wo  5:Calculate the leader’s path using FM${}^{2}$  6:Calculate first partial objectives for all follower UAVs following Equation (2)  7:**while** step<length(leaderPath) **do**  8:      Calculate the follower paths to their partial objectives using FM${}^{2}$  9:      Move all UAVs to their next partial objective 10:    leaderGoal←leaderPath(step) 11:    Calculate the next partial objectives for all follower UAVs following Equation (2) 12:    step←step+stepSize 13:**end while**

This strategy is applied to both methods proposed in the following subsections.

### 2.2. Multi UAV Coverage Path Planning Given a Target Area

Given the number of desired UAVs for the formation and a target area defined by a set of perimeter points, the proposed algorithm aims to cover the whole area while maintaining the desired flight level relative to the uneven ground level and avoiding obstacles.

**Figure 7:**
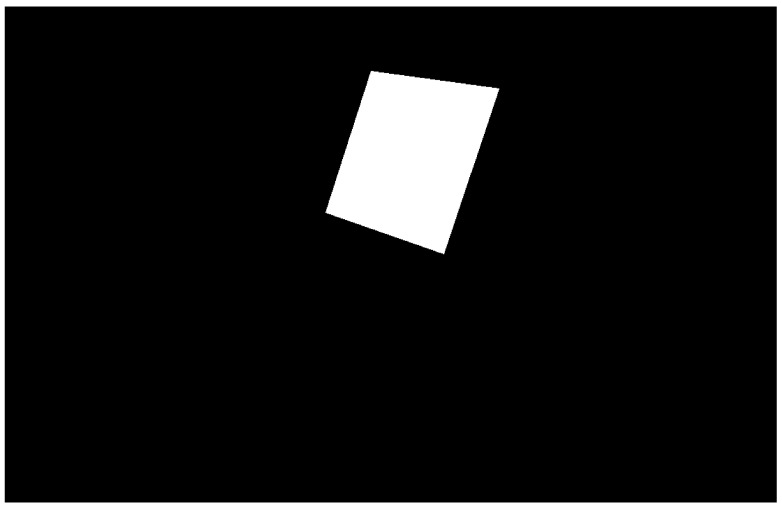
Binary mask that defines the target area to cover, defined by a given set of perimeter points.

The first step is to create a binary mask representing the target area to cover based on the given perimeter points. An example of coverage binary mask is shown in Figure 7. From now on, all figures will be trimmed down so the operations performed and the paths obtained can be seen more clearly.

**Figure 8:**
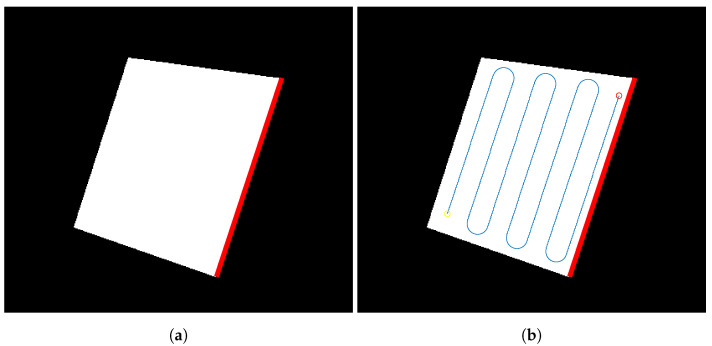
Initial 2D coverage path calculation process. (**a**) Selected best edge so the coverage path has the minimum number of turns. (**b**) Computed path using back-and-forth motions.

Then, the coverage angle is obtained by detecting the longest edge of the perimeter. This will guarantee that the coverage path has the minimum number of turns necessary. The longest edge detected for the binary mask created in the first step can be seen in Figure 8a. The path is then computed by using back-and-forth motions, with a predefined coverage bandwidth. The obtained path is shown in Figure 8b.

**Figure 9:**
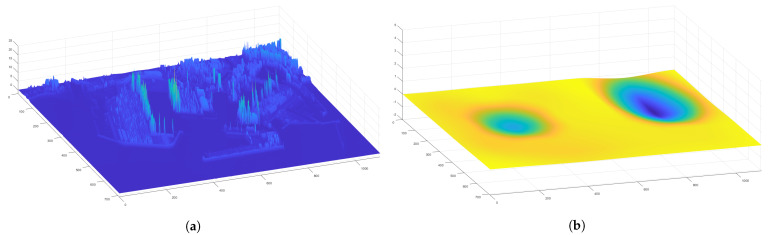
Valencia harbor environment used for the target area CPP mission. (**a**) 3D data of the Valencia harbor obtained from [46]. (**b**) Floor level data of the Valencia harbor.

Once the initial path is obtained, it is time to consider the obstacles in the environment. For that purpose, a series of processes are followed. First, a 3D map of the environment is created by using the coordinates and LiDAR data from the spanish Instituto Geográfico Nacional (IGN) [46]. An example map generated from this data is shown in Figure 9a. The data corresponds to the area around the Valencia harbor. The data obtained has a resolution of 5 m, so every cell in the 3D and 2D maps is equivalent to a 25 m${}^{2}$ area.

To compute the flight level, it is necessary to calculate a map with the floor level data. For this purpose, a grid method is used. The point with minimum height of the grid is taken as a reference, and all points of the selected grid are set to that reference height. Once the grid is moved over the whole map, a median filter and an average filter are used to smooth the obtained surface. The floor level map obtained from the Valencia harbor data is shown in Figure 9b. Note that, since the raw data is taken directly from [46], and the minimum height value in the grid is taken as the floor level value, this can lead to small errors such as negative values in the floor level map. This could be fixed by doing a more thorough post-processing of the data, but the tests have determined that this does not really affect the trajectories of the UAVs at all.

**Figure 10:**
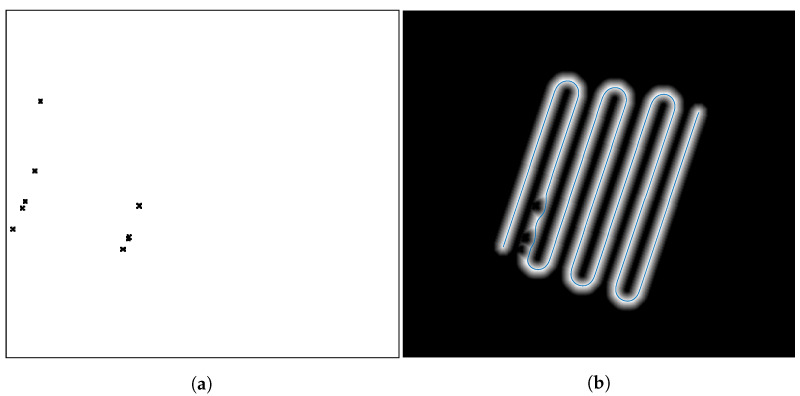
Calculation of the obstacle-free leader path. (**a**) Obstacle map calculated from the 3D data and the height of the drone paths. (**b**) Collision-free path calculated for the leader of the formation using the Fast Marching Square method.

After calculating the floor level, we can use this data and the desired relative flight level to obtain the absolute height interval of the drones performing the coverage mission. Using this interval, we can detect the obstacles inside the interval flight range of interest and discard the ones out of range. In addition, they are added to a binary mask that is used for the obstacle avoidance phase. The obstacle map is shown in Figure 10a.

When the obstacle map has been defined, the path for the leader drone is calculated with the Fast Marching Square method [47] and a binary mask created from the dilated original path and the obstacle map. The obstacles in the obstacle map are dilated to ensure a smooth collision-free path for the leader, which consequently allows for the followers to avoid obstacles using the leader-follower approach explained in the subsection above.

The obtained collision-free leader path is shown in Figure 10b.

**Figure 11:**
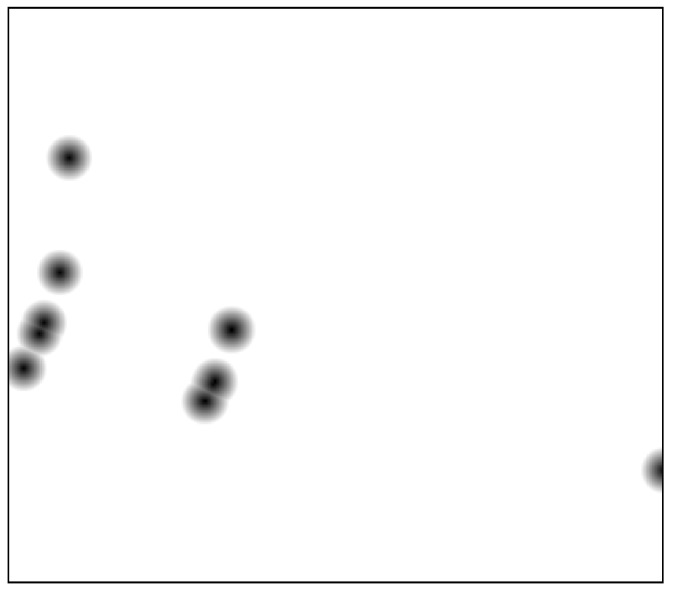
Saturated velocity map calculated from the obstacle map for the formation planning.

Next, the paths for all followers are calculated based on the collision-free leader path and the leader-follower formation strategy. One interesting feature of the velocity map is that it can be saturated to a certain value, so all vehicles traveling at a distance greater than the one specified by the saturation can travel at full speed. Figure 11 shows the velocity map obtained from the obstacle map, saturated to a value of 15 cells, equivalent to 75 m. This value is chosen so the UAVs are aware of the position of obstacles sooner, and their movement as a formation is more smooth. This also helps to set the maximum distance allowed to obstacles, which is in a range of 5–6 cells.

**Figure 12:**
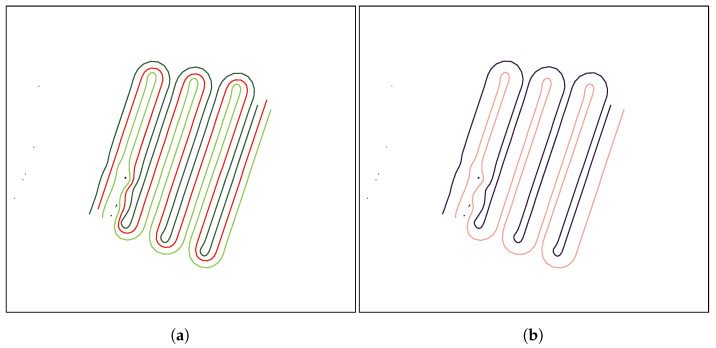
Calculation of the collision-free follower paths for teams of three and two UAVs, respectively. (**a**) Example of collision-free 2D paths using a team of three UAVs. (**b**) Example of collision-free 2D paths using a team of two UAVs.

After applying the leader-follower FM${}^{2}$ based formation approach to the leader path for teams of two and three UAVs, the 2D formation paths are obtained. Figure 12a,b shows the 2D paths for the leader and the followers for teams of two and three UAVs.

**Figure 13:**
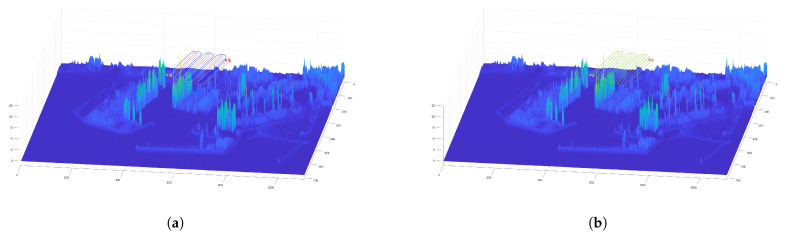
Calculation of the 3D collision-free follower paths for teams of three and two UAVs, respectively. (**a**) Example of a collision-free CPP mission given a target area using a team of three UAVs. (**b**) Example of a collision-free CPP mission given a target area using a team of two UAVs.

**Figure 14:**
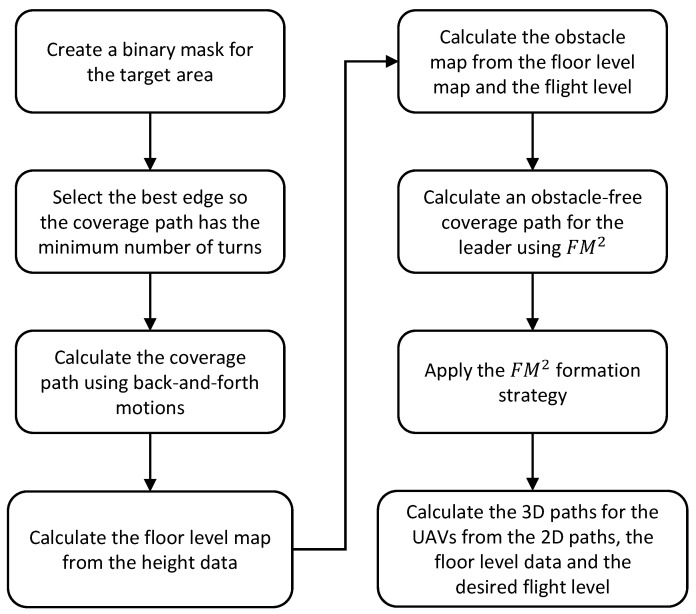
Flowchart for the CPP given a target area algorithm.

Finally, the floor level data and the desired flight level are used to add height to the paths and make them 3D. Since the floor level has been smoothed with a median and average filter, the resulting 3D paths are smooth. The obtained 3D paths are shown in Figure 13b. A flowchart showing the steps of the proposed algorithm can be seen in Figure 14.

In the next subsection, the same procedure is going to be applied to a different coverage path planning problem given a set of waypoints to cover.

### 2.3. Multi UAV Coverage Path Planning Given a Set of Waypoints

Given a number of desired UAVs for the mission and a set of waypoints they have to pass through, we propose a method to perform the coverage of all defined waypoints while avoiding obstacles.

**Figure 15:**
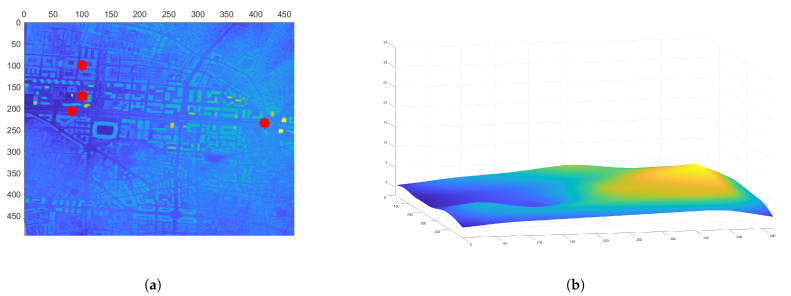
Bernabeu stadium environment used for the target area CPP mission. (**a**) Selected waypoints for the coverage mission near the Bernabeu Stadium in Madrid. (**b**) Floor level data of the Bernabeu Stadium area in Madrid.

First, the waypoints must be defined. An example of waypoints that cover a street near the Bernabeu Stadium in Madrid is shown in Figure 15a.

**Figure 16:**
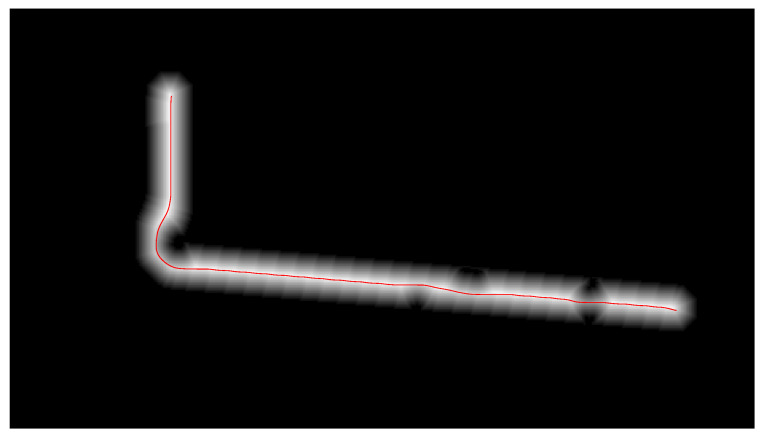
Collision-free path calculated for the leader of the formation using the Fast Marching Square method.

Once the waypoints have been selected, the procedure is the same as the followed in the previous method. An obstacle map is obtained from the floor level data (Figure 15) and the selected flight level, and an obstacle-free path is calculated for the leader of the formation. The path is calculated using the FM${}^{2}$ method, by connecting all the waypoints in order with straight lines, and then dilating them to make room for the leader UAV to maneuver and avoid obstacles. When the velocity map is created from this binary map, the generated path is smooth by nature, so the leader UAV does not necessarily pass over the exact position of the waypoint. This makes the trajectory easier to follow by a real UAV, since it resembles the operation of a pure-pursuit controller [48]. An example of an obstacle map with the corresponding path can be seen in Figure 16.

**Figure 17:**
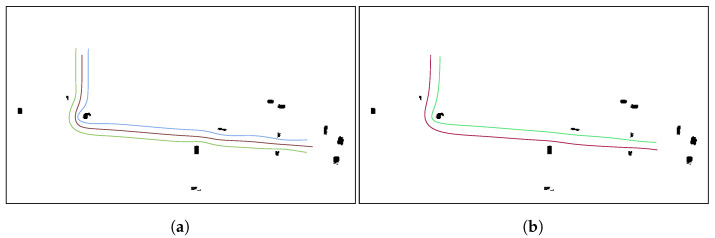
Collision-free 2D paths using teams of three and two UAVs, respectively. (**a**) Example of collision-free 2D paths using a team of three UAVs given a set of waypoints. (**b**) Example of collision-free 2D paths using a team of two UAVs given a set of waypoints.

Then, the leader-follower formation approach is applied to the generated leader’s path to create the collision free paths for the followers. The collision-free 2D paths using teams of three and two UAVs can be seen in Figure 17a,b, respectively.

**Figure 18:**
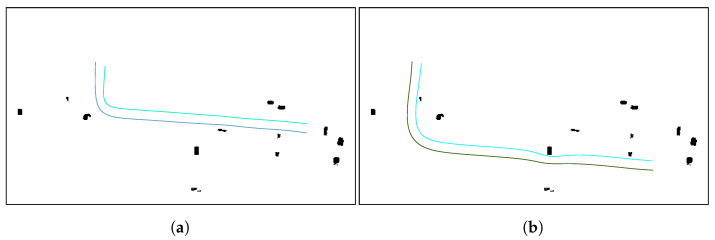
Collision-free 2D paths using teams of three and two UAVs, respectively. (**a**) Generated collision-free 2D paths using a team of two UAVs setting a certain distance to the left side of the original leader’s path. (**b**) Generated collision-free 2D paths using a team of two UAVs setting a certain distance to the right side of the original leader’s path.

Note that since the algorithm is intended to be used in urban environments, a feature has been added to generate a path that is set at a certain distance to either the left or the right of the selected waypoints, so it can be used to cover roads or busy streets while minimizing the possible risks. Figure 18a,b show the paths for the UAV formation when the distance is set to the left and right directions, respectively.

**Figure 19:**
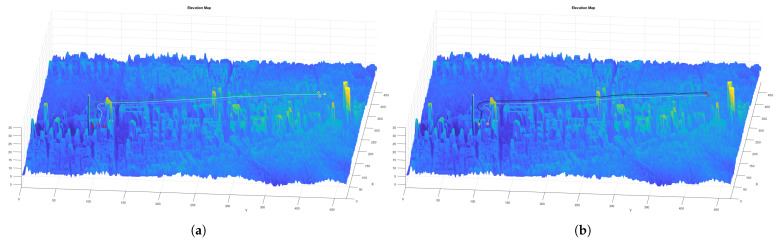
Collision-free 3D paths using teams of three and two UAVs, respectively. (**a**) Example of a collision-free CPP mission given a set of waypoints using a team of three UAVs. (**b**) Example of a collision-free CPP mission given a set of waypoints using a team of two UAVs.

**Figure 20:**
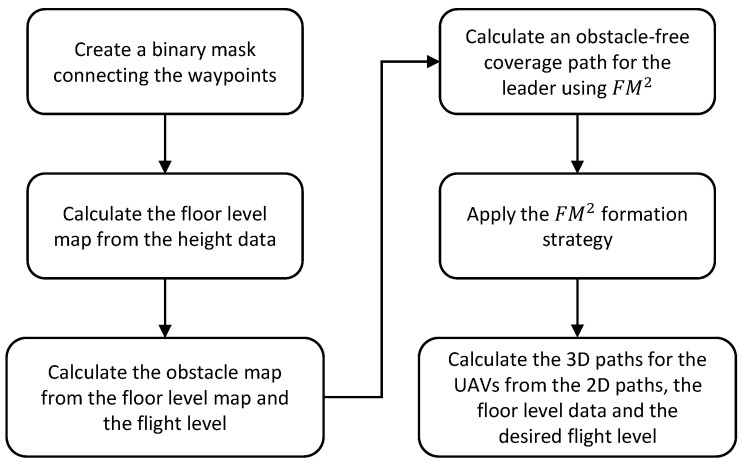
Flowchart for the CPP given a set of waypoints algorithm.

Finally, the floor level data and the desired flight level are used to add height to the paths and make them 3D. This greatly reduces the computation time, since the paths of the formation would need to be calculated in 3 dimensions, adding complexity to the algorithm. The obtained 3D paths are shown in Figure 19a,b. A flowchart showing the steps of the proposed algorithm can be seen in Figure 20.

In the next section, measures from these missions are going to be collected and analyzed to study the behavior of the algorithms, including the total execution time, the velocity map value of the follower positions in each iteration, and their distances to the leader.

## 3. Results

Once the CPP and collision avoidance formation algorithms have been implemented, some measures are taken to study the performance of the algorithms and their applicability to real world use cases. The measures taken from the experiments are the total computation time and the distance from each follower to the leader of the formation.

The machine used to run the test is a computer with 16 GB RAM and an AMD Ryzen 5 5600X CPU@4.12 GHz.

We observed that, in general, the computation times of the algorithm were high and it was not optimal to calculate every step of the formation path with the FM${}^{2}$ method if there are no obstacles around, since it only adds complexity to the computations when it is not necessary. For that, we create an hybrid approach, which only activates the FM${}^{2}$ formation algorithm when the team of UAVs is closer than a certain distance from the obstacles around it, making the calculations when there are no obstacles around much faster, since we can set the value of B (see Equation (1)) to 1 and we do not need to add all the UAVs to the velocity map and compute their paths with FM${}^{2}$.

**Figure 21:**
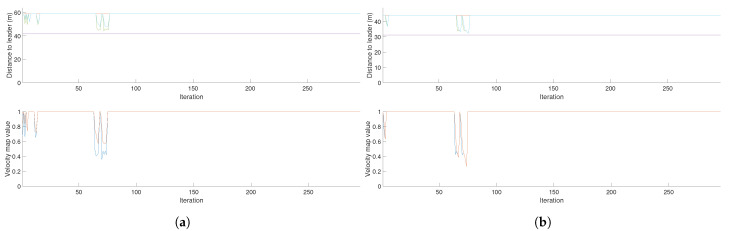
Velocity map values and distances from followers to the leader for teams of three and two UAVs performing a coverage path planning mission given a target area. (**a**) Velocity map values and distances from followers to the leader for a team of three UAVs. (**b**) Velocity map values and distances from followers to the leader for a team of two UAVs.

In the case of the CPP mission given a target area, the obtained distances to the leader and velocity map value data for a three drone mission is shown in Figure 21a.

**Figure 22:**
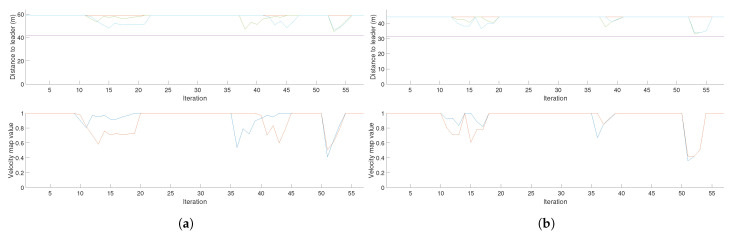
Velocity map values and distances from followers to the leader for teams of three and two UAVs performing a coverage path planning mission, given a set of waypoints. (**a**) Velocity map values and distances from followers to the leader for a team of three UAVs. (**b**) Velocity map values and distances from followers to the leader for a team of two UAVs.

The first graph shows the distance from the followers to the leader in meters. The orange line represents the reference and therefore maximum distance from the followers to the drone and the purple line represents the minimum distance. The second graph shows the value of the velocity map for the followers. In the case of the hybrid approach, this value has been set to 1 when obstacles have not been detected. As it can be seen in Table 1, the computation time is greatly decreased in the case of the target CPP area and slightly decreased in the case of the waypoints CPP. This is due to the fact that the target area CPP is conformed by more complex paths that do back-and-forth motions, so the step size to calculate the follower paths is smaller, allowing for a higher accuracy. That means that the number of iterations the algorithm ran is higher than in the case of the waypoints CPP algorithm, leading to higher computation times. Figure 21a and Figure 22a show that the target area CPP mission needs around 300 iterations, while the waypoints CPP mission uses around 60 iterations to be completed. For the case of an even number of UAVs, the distances to the leader are calculated to the virtual leader.

As the velocity map value is also a measure of the distance of the UAVs from obstacles, we can determine the minimum distance from any UAV to an obstacle based on this velocity map value and the saturation of the velocity map, which in this case is 15 cells. Since each cell has a resolution of 5 m, we can see that for the case of 3 UAVs (Figure 21a), the minimum velocity value of one follower is 0.4, which multiplied by the saturation and the resolution equals 30 m, while the other follower keeps a minimum distance of 45 m. For the case of 2 UAVs (Figure 21b) the minimum distance for both followers is approximately 22 m.

This graph also indicates that the follower UAVs do not collide with each other, as that would only happen if the velocity map value were 0, which cannot happen since the leader’s path is collision free. Based on the velocity values of both follower UAVs, the minimum distance between them is approximately 42 m in the case of the 3 UAV formation and approximately 37 m in the case of the 2 UAV formation, for the chosen coverage bandwidth of 25 cells.

Figure 21b shows the same metrics as the graph above for a team of two UAVs. Due to the multiplier value (see Equation (1)), the maximum and minimum distances from the followers are different for the two team sizes. See that Equation (1) also relates the distances from the followers to the leader to the velocity map value of the follower positions. Since they are directly correlated, the graphs show that when B decreases, the distances from the followers to the leader also decrease. In both cases, there is a disturbance at the beginning of the coverage mission, when the team of UAVs passes on the left side of the obstacles, and a second and greater disturbance when the team of UAVs passes on the right side of the obstacles at a closer distance.

In the case of the CPP mission given a set of waypoints, the obtained distances to leader and velocity map value data teams of two and three drones are shown in Figure 22.

The results obtained are similar to the case of the coverage mission given a target area. In this case, the disturbances in the formation are caused by the sets of buildings on both sides of the planned leader path. Note that, even though the leader path is the same in both cases, the formation of the three UAV team is wider than the one conformed by two UAVs. This difference can be perceived in Figure 22a,b, where the velocity map values for the team of three UAVs are lower than in the case of the team of two UAVs when passing near the same obstacles.

For the waypoint coverage mission, we can see that for the case of 3 UAVs (Figure 22a), the minimum velocity value of one follower is 0.4, which multiplied by the saturation and the resolution equals 30 m, while the other follower keeps a minimum distance of 45 m. For the case of 2 UAVs (Figure 22b) the minimum distance for both followers is approximately 37 m.

Based on the velocity values of both follower UAVs, the minimum distance between them is approximately 37 m in the case of the 3 UAV formation and approximately 35 m in the case of the 2 UAV formation, for the chosen coverage bandwidth of 25 cells.

## 4. Discussion

When solving the coverage path planning (CPP) problem, two main issues should be addressed. One of them is how to generate a coverage path that effectively covers the designated area or waypoints, and the other one is making sure that the UAV or formation of UAVs do not collide with obstacles or enter no-fly zones (NFZ). Most approaches to CPP study empty areas and focus on efficiently covering the target area and therefore use a lower resolution to reduce the computation times and make the planning easier. Some approaches do include obstacles and NFZ in their calculations, but they still use a low resolution with low cells and, in the event of having obstacles in the target area, the whole cell is marked as a NFZ and is therefore not covered. However, some methods try to reduce the effect of obstacles in their path planning by not completely avoiding zones with obstacles, but sticking to the cell’s borders.

Our proposed methods solve this issue by using the FM${}^{2}$ method to plan the trajectories. First, the obstacle-free path for the leader is calculated, and then the formation planning algorithm is used to plan the trajectories for all follower UAVs in the formation. The use of the FM${}^{2}$ method is essential for computing the leader’s path, and it is also used to make the UAV formation deformable. That means that the UAVs get closer to the leader’s path as they come closer to obstacles. The properties of the velocity map obtained with FM${}^{2}$ such as the saturation let us define the safe distance from the UAVs to the obstacles and their maximum allowable speeds.

Since the complexity of the FM${}^{2}$ algorithm can be reduced to O(n) [49], the computation times for this algorithm are really low. This allows for a fast and easy implementation of a collision-avoidance path planning algorithm with no local minima, which can be applied to any cell resolution, making it extremely useful in cluttered urban environments.

However, there are some limitations to our work. We assume that the areas where our algorithms are implemented have obstacles in determined positions but, as the first path for the leader is calculated using either back-and-forth motions or connecting the target waypoints, and then creating a binary image and dilating it to be used as a binary mask for the FM${}^{2}$. In the event of a big obstacle completely blocking the dilated path in this binary mask, the algorithm would fail to produce a valid path for the formation of UAVs. This issue can be solved by setting the velocity map cells that are not obstacles to a value slightly higher than 0, so the UAVs can move through this space out of the designated path to continue their mission, but that does not guarantee that the UAVs will continue their mission right after evading the obstacle where they left off.

Finally, practical experiments were not conducted as it is illegal to fly drones in cities without the proper licenses. Although the UAVs were tested in the simulation environments, validations in real use cases should be verified. This is left as future work when the Labyrinth project reaches its final stages.

## 5. Conclusions

The goal of this work was to design, implement, and analyze a coverage path planning formation approach with a leader-follower formation approach and FM${}^{2}$ based collision avoidance for multi-UAV teams in urban environments. The designed algorithm was implemented and its performance was analyzed through simulations.

Two coverage path planning missions were considered: one where the input is given as a target area and another one where the objective was to cover a set of waypoints. For both of them, the procedure is divided into different steps: firstly, the floor level and obstacle maps are calculated from the raw elevation data and then the collision-free leader path is calculated using FM${}^{2}$. For the target area case, a back-and-forth path is generated by drawing parallel bands to the longest edge of the target area. Secondly, the leader-follower FM${}^{2}$ formation approach is used to calculate the 2D paths for the follower UAVs. Lastly, the 2D paths get the height from the desired flight level and the obtained floor level data, generating the final 3D paths used to perform the coverage mission.

From the obtained results, we can infer that the computation times are reasonable for a mission this complex, even faster when using the hybrid approach. The distances from the followers to the leader and to obstacles have been observed to be safe thanks to the saturation levels of the velocity map and the deformable triangular formation of the team of UAVs. This approach has proven useful in cluttered urban scenarios, in line with the Labyrinth project, which financed this research and aims to integrate the safe use of UAVs in cities.

## Figures and Tables

**Table 1:** Total computation times in seconds.

	Target Area CPP		Waypoints CPP	
	2 UAVs	3 UAVs	2 UAVs	3 UAVs
Pure FM${}^{2}$	78.94	106.26	8.13	7.40
Hybrid approach	12.30	11.91	5.90	5.88

## Data Availability

LiDAR data for the environments used in this paper have been downloaded from IGN [46] http://www.ign.es/web/ign/portal (accessed on 27 April 2020).
